# Treatment of Urethral Stricture by the “Perineal Section”

**Published:** 1880-10

**Authors:** L. Ranney

**Affiliations:** Adjunct Professor of Anatomy in the Medical Department of the University of the City of New York


### ARTICLE III.

### THE TREATMENT OF URETHRAL STRICTURE BY THE "PERINEAL SECTION." BY L. RANNEY, M.D.; Adjunct Professor of Anatomy in the Medical Department of the University of the City of New York. Author of "A Treatise on Surgical Diagnosis," "The Essentials of Anatomy," etc.

(Being one of a course of lectures delivered in 1875, before the students of the Medical Department of the University of the City of New York, with some later comments.)

The operation of dividing urethral constrictions through the perineum has long been the resort of surgeons as a means of relieving symptoms, which are not only distressing to the patient but which may often endanger his life. This operation has always been considered one of difficulty, and many of the earlier surgeons, who then cut "anatomically" for the urethra, when narrowed and often-times imbedded in a mass of indurated tissue differing but little from cartilage, had every reason to approach such a procedure with caution and even fear; since the chances of failure were by no means small, if the exposing of the true urethral channel was alone counted as success. In point of fact, the establishment of a channel, no matter what was the direction or the anatomical relations of the artificial passage, so long as it ended in the bladder and would allow of the passage of an instrument, introduced at the meatus urinarius, was a surgical success of which many of the most skillful were proud. Thanks to the discovery of those small whalebone guides, which can be modified, as to the shape of the inserted end, to suit the requirements of the case or the taste of the operator, many of the dangers and difficulties of the operation are now things of the past, and a guide can thus be afforded in almost every case by which the surgeon may know when he is laying open the urethral canal or simply dividing tissues adjacent to it. I regard this discovery as one of the greatest advances in urethral surgery, of which the present century can boast; since these little instruments can be made, by skillful hands accustomed to their use, to travel the tortuous route of many an organic stricture which was once considered impermeable, and afterwards to afford a true and unerring guide for larger instruments, which are so tunneled as to follow the guide and still be capable of great latitude of movement. The soft filiform guides were but a poor substitute for these little aggressive weapons, since their flexibility was a serious obstacle to their introduction, although a possible advantage after it, and they were often worse than useless, as the skill of the surgeon and that cultivation of touch, which is so useful in the introduction of the one, were wasted in attempts to introduce the other. I have seen good surgeons play one of these deceptive instruments within the urethra of a male, while surrounding students gazed on the tiptoe of expectancy, and, when the face of the surgeon would light up with a gleam which denoted the realization of a triumph, the end of this little joker would appear at the meatus, having doubled upon itself and retraced its steps without informing the friend who had staked his faith upon it. I can conceive of no good surgeon who can to-day rely upon these guides in preference to those of whalebone, unless he be either ignorant of the various devices of which the latter are capable and the general rules for their use, or be so destitute of the perceptions of a cultivated touch that he prefers to trust to the accidental engagement of one of these soft instruments rather than to acquire the skill needed in the employment of the other.

If you will look at the specimens which I present before you, you can easily perceive that the whalebone guides are made of a diameter somewhat smaller than that of a knitting needle, and, were you all as close to me as those on the front seats, you could perceive also that each end is tapered until within a short distance of the point, when an olive shaped extremity is formed by a return to nearly the diameter of the shaft. The object of this bulb upon the point is to afford protection to the mucous membrane of the urethra, and to prevent, as far as possible, the entrance of the instrument into the lacunae of the canal, or the ejaculatory ducts.

These guides can be purchased at any of the instrument makers, or can even be made by anyone, expert in the use of an ordinary penknife, by scraping down a split piece of whalebone which can be procured in any country store. Whalebone has the advantage of flexibility, and a certain pliability when placed in hot water which admits of any modification of its form. It also never becomes brittle as does gutta-percha, which is therefore useless for such instruments. This latter property is of great benefit to the surgeon, as by use of heat, the points of these little instruments can be made to assume a bent angle, or a spiral form if demanded, and the application of cold water will make it permanent, until use has again altered it. The filiform bougie, which I have condemned, is now shown to you. It consists, as you see, of a small and very flexible guide, whose weight alone causes it to hang downwnward, as I hold it in my finger. It is used as an attachable extremity to urethral instruments, being inserted through the stricture and then attached to the end of the instrument which is destined to follow it. The extreme softness of the material of which it is made renders it incapable of doing injury, even if pushed completely into the cavity of the bladder, or of making a puncture of the urethra during its introduction, in spite of the sharpness of its point. It will be shown you in what respects the two instruments differ in their objects, when the tunneled sounds and catheters are presented to you, since, without this peculiar modification of the ordinary sound or catheter, the whalebone guide would be worthless.

I hold in my hand the ordinary sound and the tunneled instrument, in order that by seeing them together, you may have the distinctive characters of each more forcibly presented. The tunneled sound, in the first place, has a hole drilled in its point which opens upon the lower wall of the curve about one-eighth of an inch from the point, thus forming a little canal or tunnel, through which the whalebone guide can pass, if its point be introduced into it. It is important in using a tunneled instrument, that the guide, to be used, be first passed completely through the tunnel, in order to see if its diameter be not too large for the hole; and also care should be taken to guard against the edges of the orifice in the instrument being sharp, as they may cut the guide and leave a portion of the instrument within the urethra—an accident, which is of a most serious nature. In using a tunneled instrument, whether it be a sound or catheter, the guide is first passed completely to the bladder, and the protruding end of the guide is then slipped through the opening in the point of the instrument and grasped as it escapes from the opposite opening. Thus you have two instruments to handle at the same time; the first being the whalebone guide, which should remain immovable, and secondly, the tunneled sound or catheter, whose end slips along the guide as it passes down the urethral canal. The tunneled catheter is, as you see, a hollow instrument, and has its eye, through which the urine has to escape, placed somewhat farther back than in the ordinary catheter on account of the point being adapted to be used over a guide, while the sound which I have just shown you, is a solid instrument, and is designed alone as a dilator of urethral constrictions.

I have taken the opportunity of deferring the description of these instruments till the treatment of tight strictures should be reached, for the reason that they are specially designed for the relief of such cases, and are of no particular value where the caliber of the stricture will admit a No. 10 instrument; since the point, by that time, becomes so rounded and blunt as to be incapable of doing serious injury, unless force should be used, which can never make up for a lack of skill and which should always be deprecated. When I see any operator use force in urethral surgery, I invariably estimate his skill as of a low grade, no matter how high may be his standing in the profession.

There is another point of practical interest connected with tunneled instruments, which may explain why so many, who use them, make bungling work of it, and often fail in their efforts. I refer to the curve of the instrument. This curve should always be much shorter, and an arc of a much larger circle, than that of ordinary sounds, not to speak of prostatic catheters, whose curve is extreme. The reason for this alteration in the curve is this—that in all periods of the passage of the tunneled instrument, it is of vital importance that the operator be enabled to control the point of the sound or catheter without having it bind upon the guide and thus be mechanically arrested. Now when the curve of the instrument is a great one, the guide becomes so wedged against the long curve that it becomes impossible to control the point of the instrument in such a manner as to disengage the guide, and the consequence is that the guide is either cut or broken, if force be used, or that further progress of the tunneled instrument becomes impossible.

I have spent the greater portion of the hour in describing to you the instruments used in the treatment of tight strictures, and we can now intelligently study the treatment of the most severe form of stricture which the specialist meets with, viz., traumatic stricture of the deep urethra.

Strictures of this character are commonly the result of bruising of the urethra or laceration of its walls. They occur from blows received in the perineum, from falls upon some projecting bar or pointed instrument which produces injury to the deeper parts, from the injuries received during the employment of instruments by unskillful hands, from rupture of the urethra and the sloughing which usually follows it, and from transverse incisions* in the urethral walls. One of the worst strictures which I have ever met with was originally produced by a kick in the perineum, received during a quarrel; and another was produced by being impaled upon a sharp upright stick, during an attempt of the patient to jump over it. I once saw a case where the king-bolt of a cart was driven completely through the perineum into the bladder, tearing the membranous portion of the urethra and thus producing a most severe type of trouble later on.

It is the rule, after any accident which injures the urethra in its deeper portions, that the amount of induration, in the urethral walls and the tissues adjacent to the canal, becomes so extensive that all attempts at dilatation of the stricture are of no avail. Should the case happen to yield to such efforts, the constant passage of sounds at regular intervals during the life of the patient may keep the canal pervious, and thus save him the risk of a

---

* It is now well proven that transverse incisions of the urethra tend to create contraction, which is not the case with longitudinal incisions; hence a rule to be followed in all cutting operations, viz., to divide the urethra in its longitndinal axis.

cutting operation which itself usually requires subsequent care and attention to the seat of stricture to prevent its return. I never advise a patient to become the subject of the knife, whether used within or without the urethra, until the most persistent attempts at dilatation have proven unavailing; as, despite the statements of many to the contrary, I have found the dangers to the patient to be far greater when the knife was used than when the stricture was dilated.* It is for the relief then of those cases, where traumatic strictures have withstood all attempts at dilatation, that the operation which I shall now describe is indicated. I say "of traumatic strictures," since it is seldom, in my experience, that strictures not due to traumatism fail to respond to skillfully applied efforts to bring them up to the normal urethral caliber by the process of gradual dilatation.

In performing the operation of external perineal urethrotomy, with a guide,† the introduction of the whalebone director should be first passed to the bladder; and it is best to have it at least two feet in length, in order to allow of its withdrawal for a short distance, in case the progress of the staff upon it becomes obstructed by its becoming bent, or for other reasons. This same rule applies, with equal force, to all those operations within the urethra, where whalebone guides are used. This step is frequently the most difficult part of the operation, and no time should ever be begrudged, which is used in repeated attempts at introducing it.

Various devices are often required to facilitate this first step, among which may be mentioned the *bending of the point* at an *acute angle* with the shaft, so as to enable the operator to reach all points on the circumference of the urethra by simply rotating the shaft of the guide after its tip rests upon the face of the stricture; giving the inserted end a *spiral form*, so as to worm its way through a tortuous canal; and *filling the urethra* up with these guides, when, by trying different ones, the bladder may be entered and the rest then withdrawn.

---

* See my article on the dangers of urethrotomy in *N. Y. Med. Jour.*, Aug. and Sept., 1880.

† The originality of the tunneled instrument, upon which the success of this operation so much depends, is a matter of dispute between Prof. Van Buren and Prof, Gouley—both of New York City.

When the guide has been successfully passed, the patient should be anæsthetized and placed in the lithotomy position, with the wrists and ankles tied firmly together. The perineum should be then shaved, as the hairs may get between the lips of the wound and interfere with the complete union of its edges. A staff with a deep groove, and tunneled so as to be passed over the whalebone guide, is now introduced into the urethra and slid down upon the guide, as far as the anterior face of the stricture which is to be divided. This staff should be of as large a size as urethra will admit, and the groove should be sufficiently deep so as to be easily felt through the walls of the urethra, as it is a guide to the operator in dividing the urethra and thus exposing its mucous membrane and the staff which it contains.

Before the incision in the perineum is made, the staff should be entrusted to a skillful assistant, with instructions to hold it so that the groove shall be directed forward toward the operator. The surgeon should sit upon a low stool, so as to be on a plane below that of the perineum of the patient, and the external incision should be made at such a point in the median line of the perineum as to allow the urethra, when reached, to be opened slightly in front of the stricture upon the groove in the staff. The walls of the incised urethra should now be caught up and transfixed by needles threaded with strong silk, and the ends of each thread should be tied, thus forming a loop by which an assistant upon either side, can retract the urethral opening and thus expose the black whalebone guide as it lies in the urethra. The staff should now be withdrawn for a short distance, so as to more clearly expose the orifice of the stricture, and the tissues enveloping the guide should be divided with the instrument which I now hold in my hand; which, as you see, is a small blade, and, upon its point, a blunt olive pointed tip.* This knife can be crowded alongside of the guide within the grasp of the stricture, and then made to cut its way outward. A series of such cuts are often demanded to ensure the complete division of very long constrictions, and the appearance of the whalebone guide throughout the entire length of the wound tells the operator that the

* Called *a beaked bistoury.*

urethra itself has been opened. The staff is now slid along the guide, and any obstacles to its entrance into the bladder are severed; and, after it enters the bladder the staff and guide can both be withdrawn. All barriers to the escape of urine through the perineal wound should now be carefully divided, as otherwise, *pockets* may be left which shall tend to create infiltration of the urine into the surrounding parts; an accident which is liable to cause abscess and sloughing.

For the first few days, the urine will probably pass entirely through the wound in the perineum; but, as the wound tends to granulate, the escape will become gradually less, and finally cease altogether. A full sized sound should be passed, at intervals of two days, to the bladder through the meatus; the upper wall of the urethra, which is still intact, being used as a guide over which the point of the sound may be made to pass and thus be directed into the normal passage. This should be kept up until the perineal opening be entirely healed, when the patient should be taught to pass the same instrument and instructed so to do every week at least during the remainder of his life, if he wishes to guard against a recontraction of the stricture. No catheter should be tied into the bladder during the repair of the wound.

It is a point of no small importance that the scrotum should be supported, from the date of the operation until the perineal wound is practically closed, upon a broad strip of adhesive plaster extending from thigh to thigh, and notched in the centre, so as to more perfectly fit tho contour of the parts. By this step, the danger of infiltration of urine into the cellular tissue of the scrotum is avoided, and thus a serious and not infrequent complication is rendered impossible.

I have seen fit to read to you, in closing this lecture, the records of two cases from my case book, in order that the average duration of the convalescence may be appreciated, and that the latter danger, which occurred in one of them, may be impressed upon your memories. It must not be inferred, however, that all of the cases so treated recover. I think that the records of a large number of reported cases show a mortality of about five per cent.; and that rigors, abscess, and the imperfect closure of the perineal opening, make the operation one of no small danger to the patient. It is to my mind a question, however, whether the result of this method of treatment does not afford the patient a better chance of recovery than the operation of internal urethrotomy, when performed beyond the depth of *four inches* from the meatus, as the hæmorrhage, which may occur, is more easily controlled, and as the danger of extravasation, which is very great in the internal method, is of rare occurrence in the external method, if the precautions before mentioned are carefully followed.*

G. B. K., thirty-seven years of age. The patient has had one attack of gonorrhœa, some fifteen years ago, but recovered in a short time. Some twelve years ago, he fell with one leg down a coal hole and struck upon his perineum. The parts swelled rapidly and an attack of retention of urine followed, which resisted all attempts then made to relieve it, by the use of instruments. but, after the prolonged use of hot hip baths, the urine was passed spontaneously in drops. During the following two weeks, symptoms of a developing perineal abscess made their appearance, which eventually opened spontaneously, and after the pus was evacuated, the existence of a urethral fistula was discovered. This fistula healed without any special treatment, and the urinary stream gradually increased in size so that the patient has managed to empty his bladder with comparative comfort until within two years, when he began to experience marked annoyance from a constant desire to pass urine and from well marked pain at the seat of his former injury, during the act of micturition.

It was for this condition, and for a stricture which withstood all attempts of his medical attendant, that he sought my advice. An examination of the urethra shows a long and tortuous stricture, situated at six and a quarter inches from the meatus, and associated with marked induration, which could be detected through the perineum. A whalebone guide, which had been

* Since this lecture was delivered, I have endeavored to demonstrate to the profession that these views, which I then held, are sustained by the facts. In my article published in the *N. Y. Med. Journal* during the months of August and September, 1880, the deductions showed a percentage of death in the two operations to be about equal, and the ratio of complications, in the operation of internal urethrotomy, to vastly exceed those of perineal section.

given a spiral curve at its inserted end before introduction, was successfully passed to the bladder, but, as the stricture was of traumatic origin and cartilaginous in type, I deemed the operation of perineal section advisable.*

June 8th.—The operation of perineal section was this morning performed (the patient having been kept in bed for three days and my usual preparatory treatment having been followed) (see the record of following case), a staff with a deep groove being passed over a whalebone guide, which had first been introduced to the bladder, as far as the anterior face of the stricture, and the urethra being first opened upon the staff and therefore anterior to the situation of the stricture, and subsequently incised from before backward with a small beaked bistoury.

June 9th.—Through a displacement of the apparatus by which the scrotum was suspended,† a slight infiltration of the urine has taken place into the scrotum, requiring an incision to allow of a free escape of the imprisoned urine, and thus to avoid the extensive sloughing which often follows this accident, unless promptly relieved. The entire contents of the bladder passes through the wound in the perineum, requiring a sponge to be retained over that region by a "T" bandage. A full sized sound was passed to the bladder, the upper wall of the urethra being used as a guide for the instrument. Bowels moved by a dose of castor oil.

June 13th.—No change has occurred since last note, except that the wound has been granulating nicely and that sounds have been passed every other day. The temperature has not exceeded 100, nor have there been any chills or symptoms of nervous disturbance.

July 1st.—The perineal wound still remains open and a slight escape of urine is occasionally perceived from the fistulous tract. Sounds have been passed every other day and the urethra is now dilated to its full capacity. All the symptoms of infiltration of the scrotum have disappeared, and the patient is moving about the house with comparative comfort. The frequency of micturition has ceased so that the urine is retained for the normal periods.

---

* It is a well known fact in urethral surgery that strictures of a traumatic origin are seldom dilatable, and, if so, are extremely liable to rapidly return to their former condition.

† A broad strip of adhesive plaster extending from thigh to thigh beneath the scrotum.

July 15th.—The wound seems now to have completely closed, and the urine passes completely through the urethra without any apparent obstruction. Sounds are still passed at intervals, and instruction are given the patient to enable him to pass a full-sized sound to the bladder without creating irritation.

July 25th.—Patient is pronounced cured and is instructed to continue the passage of his sound at intervals of seven days, in order to prevent the return of the stricture, as a marked tendency to recontraction is usually present in cases of this character, where the induration of the urethra has been excessive and the cause of the stricture traumatic.

G. S., 27 years of age. Patient has had two attacks of gonorrhœa, but cannot definitely state the year in which they occurred. He is certain that neither lasted more than a month and that they yielded to mild injections.

About three months before he consulted me he fell across an iron bar, striking upon the perineum, but causing no injury to his testicles. An attack of retention of urine followed soon after the accident and continued for some hours, when, at the advice of his physician, he was relieved by the use of injections of hot water into the rectum and of hot hip-baths. Small clots of blood followed the first successful attempts to pass urine, and for some ten days a flexible catheter was passed to the bladder several times a day, as the bladder seemed to act feebly. At the end of this period a small tumor of the perineum appeared, which burst and left a fistulus tract through which urine escaped. A greater portion of the urine however continued to pass through the natural channel, as his physician introduced elastic bougies of gradually increasing sizes, and the patient was finally ordered by his physician to continue the use of the largest size which could then be passed. He however neglected this precaution till the stream became of extremely small size, when, becoming alarmed at the prespect of a return of the attack of retention, and finding the efforts of his physician to again dilate the stricture fruitless, l e applied to me for operation.

An examination of the urethra, at this time, showed the following condition to exist: Meatus, very small, admitting only a No. 8; a No. 4 bulbous bougie is arrested at four inches; a

No. 3 bulbous bougie is arrested at four and one-half inches; and nothing but a whalebone guide can be passed from that point to the bladder. The stream of urine is of the size of the smallest bougie, but is projected with considerable force. As the case was one which evidently demanded the operation of perineal section, and as such a step would hardly be justified without a preparatory treatment, save in cases of emergency, I ordered rest in bed, hot hip-baths morning and evening, and the internal administration of the infusion of doggrass, and the iron and bromide of potash mixture, which it is my custom to employ in such cases.*

June 10th.—A large dose of castor oil is given to day, preparatory to the operation, as the patient has now had four days of confinement to bed and the other steps of preparation have been faithfully carried out. A second examination of the urethra to-day reveals very marked induration in the vicinity of the peno-scrotal junction and extending backward into the perineum.

June 11th.—After anæsthetizing the patient, a whalebone guide was passed without much difficulty at the second attempt, a spiral curve being given to the end of the instrument. The meatus required division, in order to allow of the introduction of a tunneled staff of sufficiently large size to be easily felt in the perineum, and a staff corresponding to a No. 10 sound was then passed down till it rested upon the anterior face of the stricture. It was found that the first incision would have to be started about one inch higher up than is usually required for strictures of the deep urethra, in order to open the urethra in front of the stricture, where the groove on the staff acted as the guide to the operator. No special point of interest presented itself save the difficulty in dividing the indurated tissues and exposing the black whalebone guide, which were extremely dense and rendered the wide separation of the edges of the wound difficult. After completely dividing the stricture, the staff was slid along the guide and all obstacles to its free entrance into the bladder divided.

---

* The infusion of doggrass (Triticum Repens) was first brought to my notice by Prof. Gouey, who had used it for some time as a diuretic. It is given in large doses, and as much as a pint or two being given in twenty-four hours The other mixture mentioned is as follows:

R. Tr. Ferri Mur. one ounce; Sol. Pot. Brom. (saturated) three ounces; Sig. one drachm in water, after meals. It acts as a tonic and diuretic, and also exerts a special anæsthetic influence on the urinary passages.

The tunneled staff and its guide were then withdrawn, a full sized sound was passed without any obstruction being detected.

June 12th.—Pulse 100; temperature 99¼; respiration 20. One ounce of urine passed through the urethra during the night. General condition excellent.

June 13th.—Full sized sound passed to bladder. A slight hæmorrhage from the perineal opening occurred last night, which was arrested by digital compression over the wound. Temperature 99.

June 14th.—No urine has passed through the penis since the first night after the operation, probably on account of the swelling of the tissues. The patient has no evidences of fever, and passes urine only three or four times during the twenty-four hours.

June 18th.—Urine now begins to escape by the natural channel; the wound is granulating rapidly. Sounds are passed every other day.

June 19th.—Patient passes almost all of the urine by the urethra, and is able to sit up all day in an easy chair.

June 26th.—Since the last note, the perineal wound has been fast closing, and now little if any urine escapes in that direction. The strength is fast approaching the normal standard and the patient walks about without discomfort.

July 15th.—To-day the patient demonstrated his ability to pass a large sized sound upon himself and is pronounced cured. He is ordered to continue the use of instruments, at intervals of a week, for the rest of his life, to prevent reconstruction of the stricture.

